# Pathological alleles of MPV17 modeled in the yeast *Saccharomyces cerevisiae* orthologous gene *SYM1* reveal their inability to take part in a high molecular weight complex

**DOI:** 10.1371/journal.pone.0205014

**Published:** 2018-10-01

**Authors:** Micol Gilberti, Enrico Baruffini, Claudia Donnini, Cristina Dallabona

**Affiliations:** Department of Chemistry, Life Sciences and Environmental Sustainability, University of Parma, Parma, Italy; University of Alabama at Birmingham, UNITED STATES

## Abstract

Mitochondrial DNA depletion syndromes (MDDS) are a genetically and clinically heterogeneous group of human diseases caused by mutations in nuclear genes and characterized by a severe reduction in mitochondrial DNA (mtDNA) copy number leading to impaired energy production in affected tissues and organs. Mutations in the *MPV17* gene, whose role is still elusive, were described as cause of the hepatocerebral form of MDDS and Navajo neuro-hepathopathy. The high degree of conservation observed between *MPV17* and its yeast homolog *SYM1* made the latter a good model for the study of the pathology. Here, we used *Saccharomyces cerevisiae* to elucidate the molecular consequences of seven *MPV17* missense mutations identified in patients and localized in different protein domains. The phenotypic analysis of the appropriate *sym1* mutant strains created demonstrated deleterious effect for all mutations regarding OXPHOS metabolism and mtDNA stability. We deepened the pathogenic effect of the mutations by investigating whether they prevented the correct protein localization into the mitochondria or affected the stability of the proteins. All the Sym1 mutant proteins correctly localized into the mitochondria and only one mutation predominantly affects protein stability. All the other mutations compromised the formation of the high molecular weight complex of unknown composition, previously identified both in yeast, cell cultures and mouse tissues, as demonstrated by the consistent fraction of the Sym1 mutant proteins found free or in not fully assembled complex, strengthening its role as protein forming part of a high molecular weight complex.

## Introduction

Mitochondrial DNA depletion syndromes (MDDS) are a genetically and clinically heterogeneous group of human diseases caused by mutations in nuclear genes [1,2]. Despite the very different clinical manifestations of these severe diseases, all are characterized by profound reduction in mitochondrial DNA (mtDNA) copy number in one or several tissues, leading to impaired energy production in affected tissues and organs [3]. So far, mutations in at least 16 nuclear genes have been associated with low copy number of mtDNA within cells [2–4]. These includes *TK2*, *TYMP*, *DGUOK*, *RRM2B*, *SUCLA2*, *SUCLG1*, *ABAT*, *AGK*, *POLG*, *TWNK*, *TFAM*, *MGME1*, *RNASEH* genes involved in mtDNA maintenance, either by controlling the supply of deoxyribonucletides for, or by carrying out, the synthesis of mtDNA. Besides mutations in these well-characterized genes, mutations in the *MPV17* gene were described as cause of hepatocerebral MDDS [5] and Navajo neuro-hepathopathy [6]. The human *MPV17* gene, located on chromosome 2p23-21, encodes a small protein of 176 amino acids [7] characterized by four predicted transmembrane spans and located in the inner mitochondrial membrane [5]. Since then, more than 30 different *MPV17* mutations were identified confirming these inherited autosomal recessive mutations as prominent cause of hepatocerebral MDSS, accounting for about 50% of the cases (S1 Table). However, the functional link between Mpv17 and mtDNA maintenance is still elusive.

A high degree of conservation has been observed between *MPV17* and its mouse (*MPV17*), zebrafish (*tra*) and yeast (*SYM1*) homologs, although mutants in these genes show very different phenotypes [8]. The *Saccharomyces cerevisiae* ortholog Sym1 is a heat-induced protein [9], required for OXPHOS metabolism in stress conditions (high temperature, high ethanol concentration), with a role in controlling the flux of Krebs cycle intermediates across the inner mitochondrial membrane [10]. In addition, point mutations equivalent to those found in patients affected by MDDS, cause mtDNA instability, leading to increased accumulation of mitochondrial respiratory deficient “petite” mutants [5].

In yeast, studies based on blue native gel electrophoresis have demonstrated that Sym1 takes part within a high molecular weight complex >600 kDa, the composition of which is, however, unknown [10]. By reconstitution into lipid bilayers, Sym1 has been confirmed to aggregate in a high molecular weight complex to form a membrane pore in the inner mitochondrial membrane, whose dimension is sufficient to allow the transport of large molecules such as metabolites across the inner mitochondrial membrane [11]. The role of Mpv17 as a Δψm-modulating channel, that apparently contributes to mitochondrial homeostasis under different conditions, was more recently demonstrated also for the human protein [12]. However, the physiological role of the channel and the nature of the cargo remain elusive.

In zebrafish, the loss of tra results in a severe decrease of iridophores [13]. As these cells have a special requirement of guanine, it has been suggested that iridophores death in tra mutant might be the result of mitochondrial dysfunction, consistent with a defect in the import of either dGTP or its precursors. Deoxynucleotides insufficiency was more recently demonstrated in Mpv17^-/-^ mice and in quiescent fibroblasts of patients carrying mutations in *MPV17* suggesting dNTP insufficiency in the mitochondria as the cause of mitochondrial DNA depletion in *MPV17* deficiency [14].

Notably, in both cell cultures and mouse tissues, Mpv17 is part of a high molecular weight complex of unknown composition, which is essential for mtDNA maintenance in critical tissue, i.e. liver, of a *MPV17* knockout mouse model [15]. The findings that in both cell cultures and mouse tissues Mpv17 is part of a complex, like the ortholog Sym1 in yeast, confirm yeast as a good model for the study of the molecular mechanisms underlying the pathology. The effects of seven point mutations, equivalent to those found in MDDS patients, were here analyzed in *Saccharomyces cerevisiae* for their effect on mitochondrial functionality, mtDNA stability, protein stability, localization and capability to take part to a high molecular weight complex. Here we demonstrated that only one mutation predominantly affects protein stability, whereas all the other mutations compromise the inclusion of Sym1 in the complex strengthening its role as protein forming part of a high molecular weight complex.

## Materials and methods

### Yeast strains, plasmids and media

The yeast strain used in this study was BY4741 *sym1*::*kanMX4* (*MATa; his3Δ1 leu2Δ0 met15Δ0 ura3Δ0 sym1*::*kanMX4*).

The pFL38-*SYM1* [10] vector was modified in order to add the HA-tag on the C-terminus just before the stop codon. *SYM1-HA* was mutagenised by PCR overlap [16] with appropriate primers (S2 Table) to obtain the allelic variants *sym1*^*G24W*^*-HA*, *sym1*^*R51Q*^*-HA*, *sym1*^*R51W*^*-HA*, *sym1*^*P104L*^*-HA*, *sym1*^*A168D*^*-HA*, *sym1*^*N172K*^*-HA*, *sym1-*^*S176F*^*-HA* and cloned into the pFL38 vector.

The strain BY4741 *sym1*::*kanMX4* was transformed through the Li-Ac method [17] with the empty centromeric vector pFL38 [18] or the vector carrying the *SYM1-HA* tagged wild type allele or the mutant tagged alleles.

Cells were cultured in synthetic complete media SC (0.69% YNB without amino acids powder, ForMedium, supplemented with 1g/l dropout mix [19] without uracil to keep the pFL38 plasmid) or in YP (1% Yeast extract, 2% Peptone, ForMedium). Media were supplemented with various carbon sources at various concentrations as indicated, in liquid phase or after solidification with 20g/l agar (ForMedium).

### Mitochondrial DNA mutation frequency

Strains were pregrown at 28°C in SC medium supplemented with 2% ethanol in order to counterselect the *petite* cells that could be present in the population and then inoculated in SC supplemented with 2% glucose and incubated for 4 h at 28°C. Due to the role of Sym1 both in heat shock and in ethanol tolerance [9], to enhance the mutant phenotype, 2% ethanol was added and the cultures were shifted at 37°C. After 15 generations of growth at 37°C, cells were plated on SC agar plates supplemented with 2% ethanol and 0.25% glucose at a dilution giving approximately 200 cells/plate. *Petite* frequencies were defined as the percentage of colonies showing the *petite* phenotype after 5-day of incubation at 28°C.

### Isolation of mitochondria and protein extraction

Cells, pregrown at 28°C in SC supplemented with 2% ethanol, were exponentially grown in SC supplemented with 2% glucose at 28°C and transferred to SC medium supplemented with 0.6% glucose plus 2% ethanol at 37°C for 14 h.

To determine the cellular localization (mitochondrial vs cytosolic) of the Sym1 wild type or mutant proteins we have extracted mitochondrial and cytosolic protein fraction as previously reported [20] with minor modifications. Briefly, cell wall was removed using digestion buffer containing 1.2M sorbitol, 60mM K-phosphate pH 7.5, 1mM EDTA, 1% β-mercaptoethanol, 1mg/ml Zymolyase-20T (Nacalai tesque) to obtain spheroplasts. Spheroplast were homogenized by glass/Teflon potter using 30 gentle strokes on ice. The supernatant containing the cytosolic fraction was separated from mitochondrial pellet by centrifugation.

To determine Sym1 protein stability we have performed total protein extraction with the trichloroacetic acid (TCA) method by chilling the cells supplemented with 120 mM NaOH, 0.5% β-mercaptoethanol, 650 μM PMSF and 25% TCA on ice, then re-suspending the proteins in Laemmli sample buffer at pH 6.8.

Mitochondrial proteins for BN-PAGE analyses were obtained by suspending the cells in extraction buffer containing 0.6 M sorbitol, 10 mM imidazole, 0.5 mM EDTA, 0.1% BSA and 1mM PMSF. Cells were broken by vortexing on ice using glass beads and mitochondrial proteins were obtained by centrifugation and re-suspended in the extraction buffer.

Quantification of protein concentration was performed by Bradford’s method [21] using Bio-Rad protein assay following the manufacturer’s instructions.

### Gel electrophoresis and western blot analysis

Total protein extract or mitochondrial protein extract was load on 12% SDS–polyacrylamide gel and Western Blot was performed.

For two-dimensional BNGE, 150 mg of protein from isolated mitochondria was treated as previously described [22]; then the samples were loaded and run into a 3–11% gradient non denaturating 1D-BNGE. The denaturing 2D-BNGE electrophoresis was performed as previously described [10].

Gels were electroblotted onto nitrocellulose filters and sequentially immunostained with specific antibodies against HA (Roche Applied Science), Por1 and Pgk1 (Abcam Mitoscience). After incubation with the appropriate secondary antibodies, ECL Western blotting Substrate (Clarity^TM^, BioRad) was used for final detection. Signals were quantified through Quantity One Software (Bio-Rad).

### *In silico* prediction of Sym1 mutant protein stability

Protein stability was predicted using four prediction tools: I-mutant 2.0 (http://folding.biofold.org/cgi-bin/i-mutant2.0.cgi), Mupro (http://mupro.proteomics.ics.uci.edu/), Istable (http://predictor.nchu.edu.tw/istable/) and INPS-MD (https://inpsmd.biocomp.unibo.it/welcome/default/index). In all cases, default parameters were maintained, except for temperature which was set at 37°C.

### RT-qPCR

Total RNA was extracted from cells grown in the same condition as for protein extraction. For the reverse transcription, total RNA was treated with DNase I (New England Biolabs), retro-transcribed with M-MuLV Reverse Transcriptase (New England Biolabs) with oligo (dT)_20_ primer (Euroclone) and murine RNase inhibitor (New England Biolabs). qPCR on retro-transcribed *SYM1* and, as reference, *ACT1* was performed by using Power Sybr Green mix with ROX (Kapa Biosystems), supplemented with primers qSYM1Fw and qSYM1Rv or ACT1qFw and ACT1qRv, at a final concentration of 120 nM in the AB 7300 (Life Technologies) instrument at default settings: 50°C 2 min, 95°C 10 min, 41 cycles at 95°C for 15 s and 60°C for 1 min, and one cycle at 95°C for 15 s and at 60°C for 15 s. Statistical analysis was performed through an unpaired two-tailed t-test.

## Results

### Yeast model

To evaluate if the mutations have a detrimental effect on yeast mitochondrial functionality, we created constructs carrying the *SYM1* sequence, wild type or mutant versions, added of the haemagglutinin (HA) epitope coding sequence. The tagged gene was under the control of the natural *SYM1* promoter and terminator. The presence of the tag, necessary for immunodetection assay as there is no effective anti-Sym1 antibody, does not prevent the correct mitochondrial localization nor compromise the presence of Sym1 in a high molecular weight complex [10]. The previously used construct did not possess the complete *SYM1* terminator sequence, that here has been added because in its presence an increased protein production was observed (data not shown). Firstly, we have tested the functionality of *SYM1-HA* construct determining its ability to complement the *sym1Δ* OXPHOS defective phenotype and mtDNA instability. Although the null mutant transformed with the untagged *SYM1* showed a little bit better performance regarding these two phenotypes respect to the strain transformed with *SYM1-HA*, the rescue of the defects by the latter construct is fully evident (data not shown).

### Phenotypic analyses

**Fig 1:**
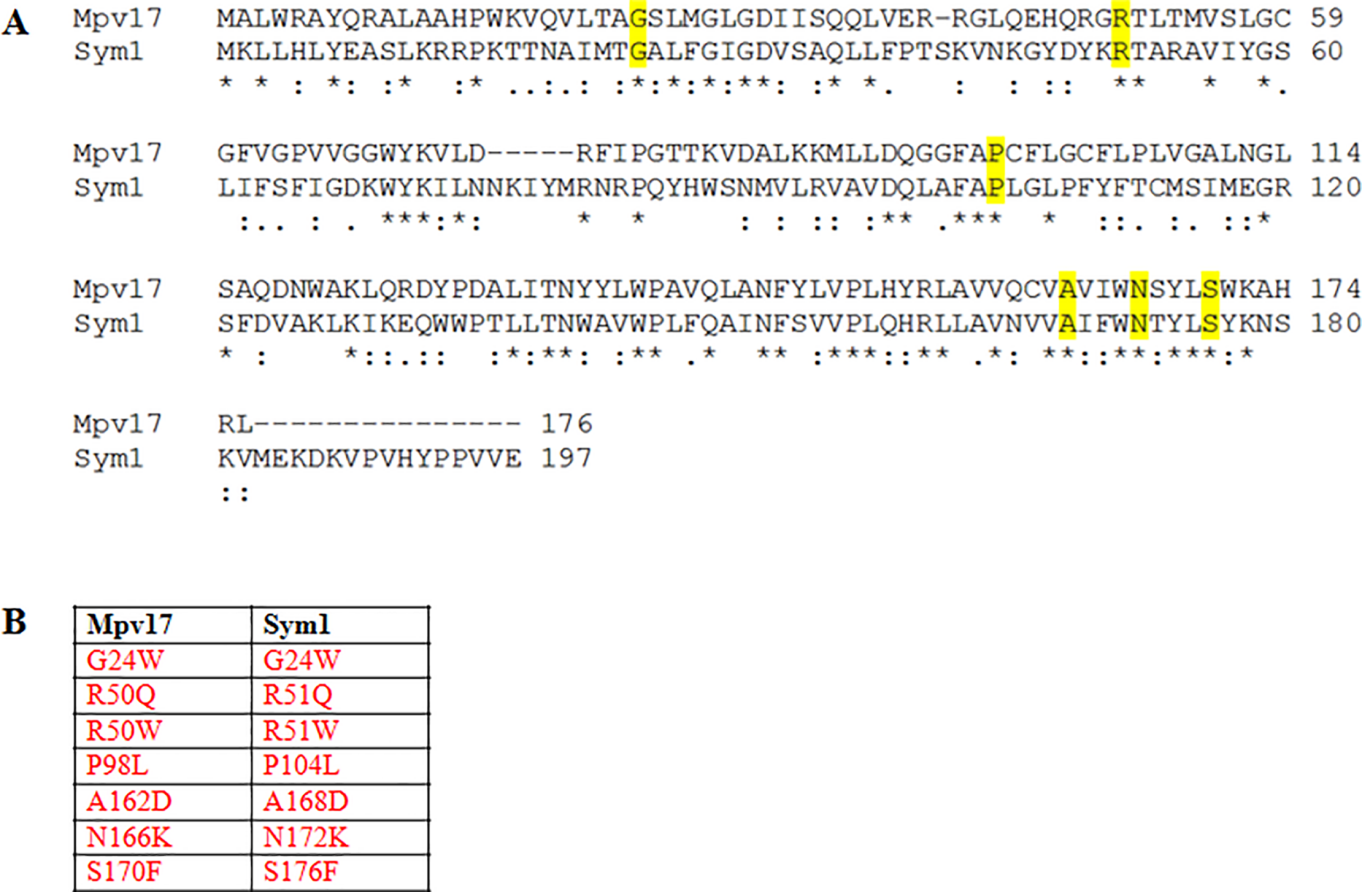
Protein alignments and missense mutations. A) CLUSTAL Omega alignments of the Mpv17 and Sym1 proteins. The missense mutations conserved between the two proteins and analysed are shown in yellow. B) List of missense mutations studied and conserved between Mpv17 and Sym1 proteins.

**Fig 2:**
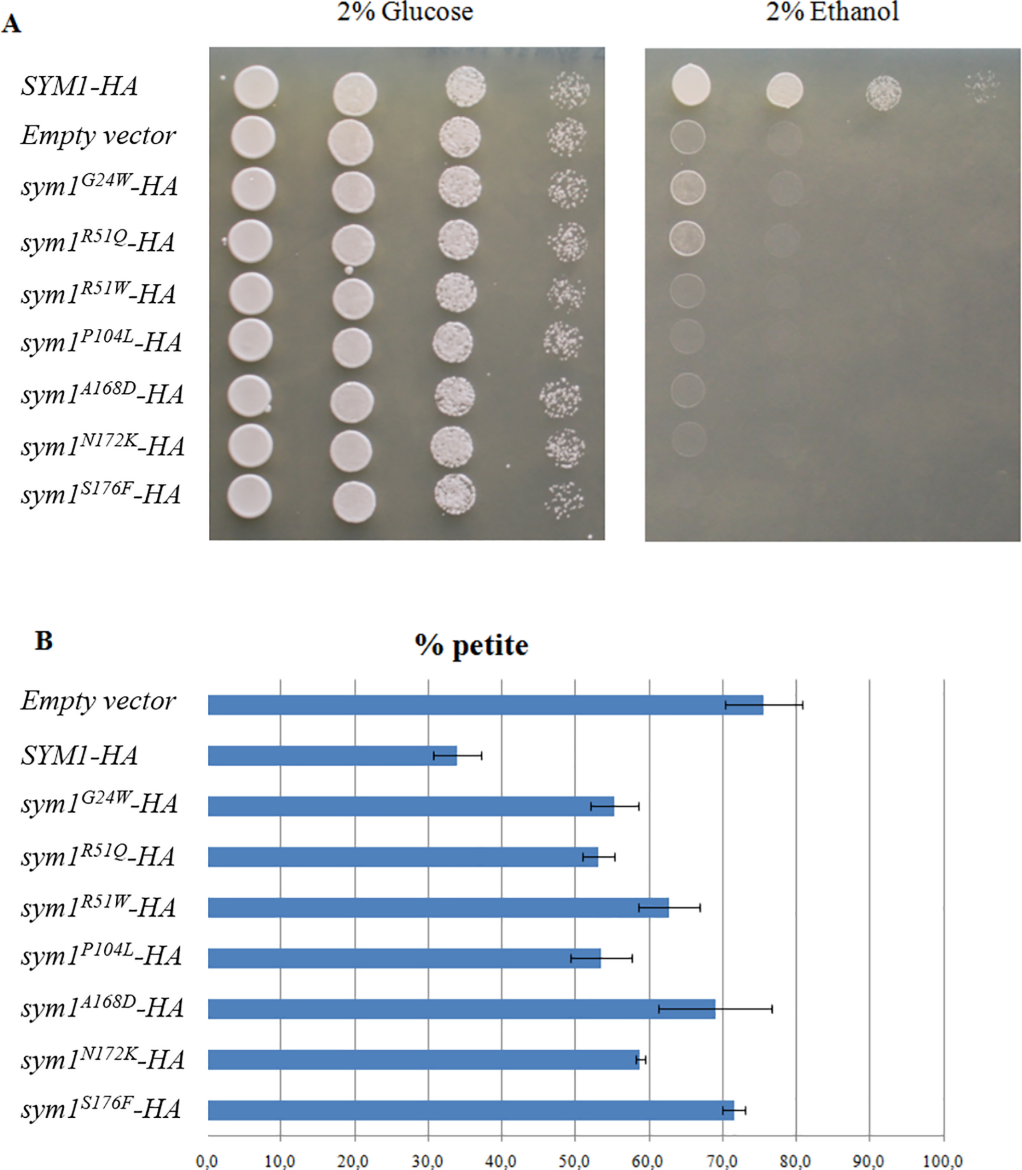
Phenotypic analysis. (A) Oxidative growth phenotype at 37°C. Equal amounts of serial dilutions of cells from exponentially grown cultures were spotted (5x10^4^, 5x10^3^, 5x10^2^, 5x10^1^ cells/spot) onto YP plates supplemented with the indicated carbon sources. Growth was scored after 3 days of incubation at 37°C. (B) Respiratory-deficient mutants (*petite*) accumulation after 15 generations of growth at 37°C. Blue bars represent the percentage of *petite* More than 4000 colonies per strain were scored. All values are means of three independent experiments. The error bar represents the standard deviation.

Nonsense and missense mutations, deletions and insertions, have been identified in coding and splicing region of the *MPV17* gene in a variety of patients (S1 Table). Among human mutations in amino acids conserved between hMpv17 and ySym1, we studied the pathogenic role of seven ones (Fig 1) on mitochondrial function introducing the corresponding mutant alleles into the BY4741 yeast strain disrupted in *SYM1* (*sym1Δ)*. We first tested the possible deleterious impact of the alleged pathogenic mutations on the OXPHOS phenotype. The temperature-sensitive OXPHOS phenotype of the *sym1* null mutant was rescued by re-expressing the wild-type *SYM1* gene fused in frame with the hemagglutinin tag (*SYM1-HA*). On the contrary, the growth of all the strains transformed with mutant *Sym1-HA* alleles was impaired in medium containing 2% ethanol as the sole carbon source at 37°C (Fig 2A), as previously demonstrated for three of the mutations under consideration [5]. The mutations R51W (hR50W), P104L (hP98L), A168D (hA162D), N172K (hN166K), S176F (hS170F), resulted in a severe oxidative growth defect, comparable to that of the *sym1Δ* strain, whereas the G24W (hG24W) and R51Q (hR50Q) mutations determined a relatively milder OXPHOS phenotype compared to the *sym1Δ* strain, although the oxidative growth was severely compromised.

We also determined the effect of the mutations on mtDNA stability measuring the frequency of mitochondrial respiratory deficient mutants (*petite*). The rationale for *petite* formation is that colonies generated by respiratory deficient cells, which have lost mtDNA or have large deletion on it, are smaller than the colonies generated by respiratory proficient wild type cells on media containing a limiting amount of fermentative carbon sources, because they are unable to produce biomass through respiration of ethanol, the end product of fermentation. *Petite* cells arose spontaneously in wild type strains at high frequency (around 1–10% depending on the strains) and allow to assess the effects of a mutation on mtDNA stability [23]. The *sym1Δ* strain showed a significant increase of *petite* mutants compared to the *SYM1* parental strain; the re-expression of the *SYM1-HA* gene was able to reduce the *petite* frequency (Fig 2B). All strains expressing the mutant variants showed a significant increase of *petite* colonies compared to the wild type, indicative of a defective mtDNA maintenance.

The phenotypic analyses demonstrate the deleterious effect of the seven missense mutations studied and confirmed a role of Sym1 in OXPHOS metabolism and mtDNA stability.

### Localization and levels of the mutated Sym1 proteins

**Fig 3:**
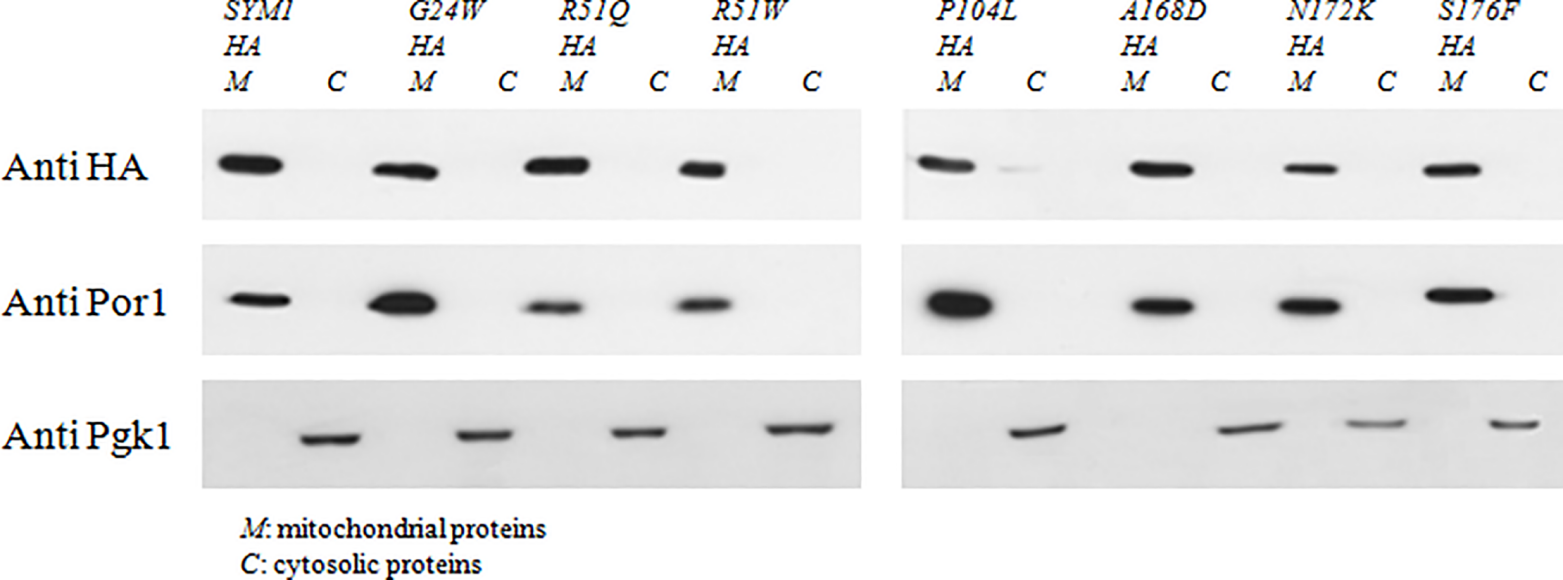
Proteins localization. Western blot on denaturing SDS–PAGE of mitochondrial and cytosolic proteins from *sym1Δ* strains expressing the HA-tagged Sym1 recombinant variants. An antibody against HA-tag was used to detect the tagged Sym1 recombinant variants; antibodies against Por1 and Pgk1 were used as controls.

To deepen the pathogenic effect of the mutations, we investigated whether they prevent the correct protein localization into the mitochondria, taking advantage of having strains expressing HA-tagged *SYM1* recombinant variants. Cytosolic and mitochondrial protein fractions were extracted as described in Material and Methods, from cells grown in conditions in which the percentage of *petite* colonies was comparable between the *sym1Δ* mutant and the parental *SYM1* strain. The proteins were separated by gel electrophoresis and analyzed by Western blot, and the Sym1 proteins were detected using the antibody against the HA tag. Antibodies against porine (Por1) and phosphoglycerate kinase (Pgk1) were used as markers for the mitochondrial and cytosolic fractions respectively. All the Sym1 mutant proteins, beside the wild type one, correctly localized into the mitochondria as demonstrated by the immuno-signal in the mitochondrial fraction (Fig 3).

**Fig 4:**
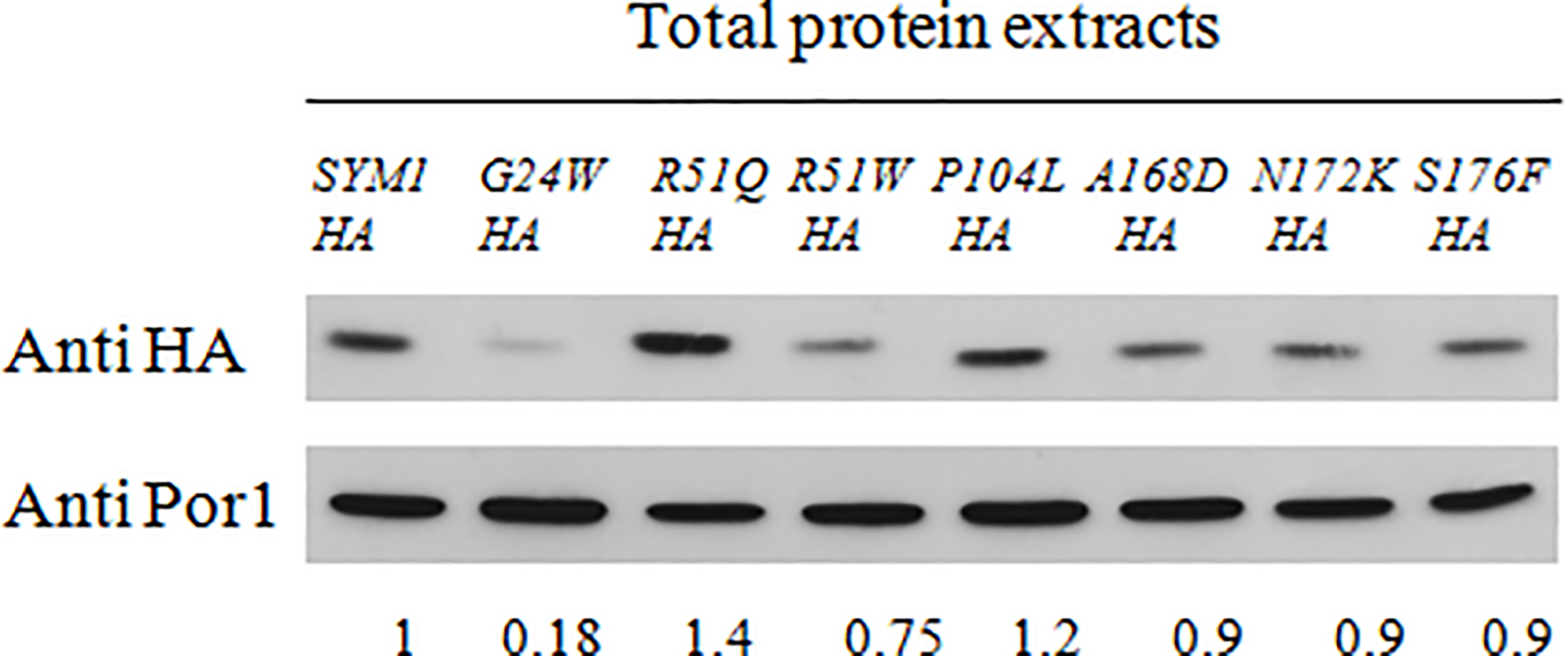
Proteins stability. Western blot on denaturing SDS–PAGE of proteins from *sym1Δ* strains expressing the HA-tagged Sym1 recombinant variants. An antibody against HA-tag was used to detect the tagged Sym1 recombinant variants and an antibody against Por1 was used as a loading control. The quantification was performed using the Quantity One software. Normalization was performed and the value 1.0 was attributed to the wild type.

To check whether the mutations affected the stability of the proteins, we determined the steady-state level of the Sym1 mutated proteins by Western blot analysis on mitochondrial protein fraction. We quantified the amount of the seven mutant proteins compared to the wild type and we found that the protein carrying the G24W mutation showed a drastic amount reduction; instead, the proteins carrying the R51W, A168D, N172K and S176F mutations showed a slight amount reduction. No difference was observed for the R51Q and P104L mutations (Fig 4). An overexposure of the signals showed a degradation band (containing the C-terminal HA tag) specific for the *sym1*^*G24W*^*-HA* mutant strain, suggesting that this protein is unstable and degraded (S1 Fig). A further overexposure for longer times showed further bands neither in the *sym1*^*G24W*^*-HA* mutant strain nor in other mutant strains. We also performed an *in silico* test using four different prediction tools (I-mutant 2.0, Mupro, Istable and INPS-MD) to evaluate whether the presence of this mutation in *MPV17* is predicted to alter protein stability. Other two mutations, R50Q (yR51Q) and R50W (yR51W), were evaluated, since associated to a normal protein level and to a slightly reduced level, respectively. Although the information obtained by this method is not completely reliable since not based on the protein structure, it is interesting to observe that three of the tools predict a decreased stability for the G24W mutant protein (S3 Table).

Finally, in order to investigate whether the differences observed at protein level were due to any problem affecting transcription, the transcript levels of *SYM1* wild type and mutant genes were assessed by RT-qPCR. Cell were grown in the same condition used for the protein stability analyses, cDNAs were synthesized and quantitative PCR was performed. The RNA levels of the *SYM1* genes were normalized to *ACT1* levels, used as internal control. The mRNA levels of *sym1* mutant genes were increased compared to wild type, except for the mRNA of *sym1*^*S176F*^ mutant gene which showed expression levels comparable to that observed for wild type gene (S2 Fig). The increase of mRNA levels suggests that the unfunctional proteins induce a feedback effect increasing the gene transcription. However, this increase is not associated with a higher protein quantity respect to the wild type, highlighting instability in most of the mutated proteins. In the strain carrying the G24W mutation the protein quantity observed was very low confirming a high instability of this mutant protein.

### Presence in a high molecular weight complex of Sym1 protein

**Fig 5:**
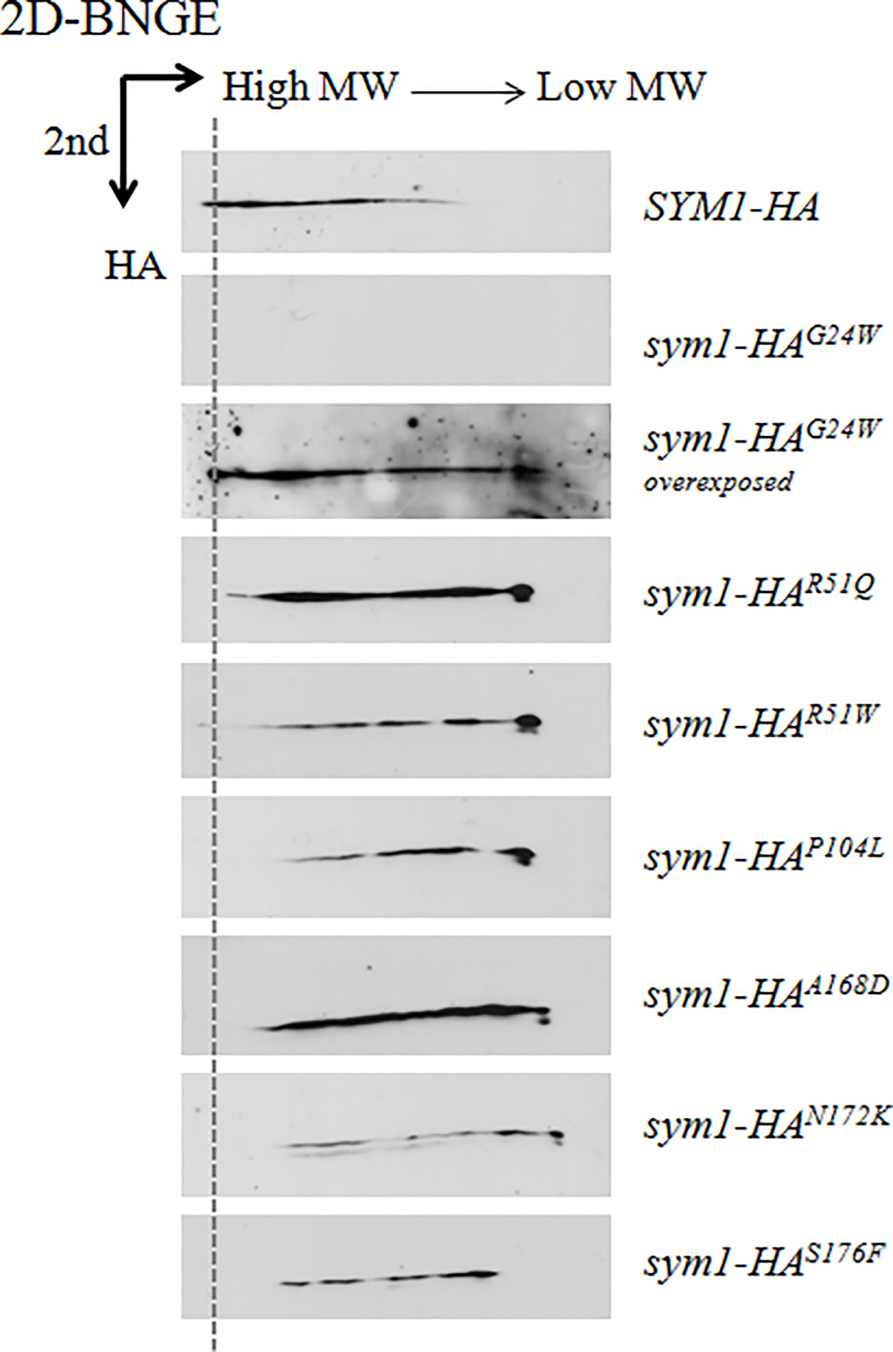
High molecular weight complex detection. Western blot of second dimension (2D) blue-native gel electrophoresis of mitochondrial proteins from *sym1Δ* strains expressing the HA-tagged Sym1 recombinant variants. An antibody against HA-tag was used to detect the tagged Sym1 recombinant variants.

As previously described, Sym1 takes part in a high molecular weight complex [10] as represented by dashed line in Fig 5. To better understand the molecular mechanisms underpinning the pathology, we have also investigated whether the seven mutations prevented the Sym1 capability to take part in the fully assembled complex. We carried out 2D-BNGE analysis on mitochondrial proteins extracted from strains expressing the HA-tagged Sym1 recombinant variants. All the mutations, although to a different extent, compromised at least partially the Sym1 interaction with the other complex components, giving rise to a not fully assembled complex, as shown by the signals of the mutated proteins that are shifted in the low molecular weight side of the gel (Fig 5). In addition, a consistent fraction of the Sym1 mutant proteins was found free. Furthermore, according to the reduction in protein stability induced by the G24W mutation, the Sym1^G24W^ mutant protein detection needed a longer exposure time than that required by the other mutant proteins.

## Discussion

The deletion of *SYM1* in *S*. *cerevisiae* being complemented by the expression of the human *MPV17* gene and the absence of *SYM1* functional paralogues have established Sym1 as a good model for the study of Mpv17 function. In yeast, another protein shows sequence similarity to Mpv17, Yor292c. This protein was localized in the vacuole in a large-scale study [24]. Furthermore, experiments performed in our laboratory (Dallabona, unpublished data) did not reveal any role of this protein on mitochondrial function, as its open reading frame deletion did not affect oxidative growth nor mtDNA stability. Furthermore, the *sym1Δ* phenotype is not worsened by YOR292c deletion, suggesting that the function is not redundant.

Here, we used the yeast *S*. *cerevisiae* to elucidate the molecular consequences of a set of *MPV17* missense pathological mutations identified in MDDS patients and localized in different domains of the Sym1 protein. It should be noted, however, that although *in silico* analyses show that the architecture of the transmembrane helices of Sym1 and Mpv17 is highly conserved, with a proposed topology of four transmembrane spans in an N_out_/C_out_ orientation [11], the bioinformatic programs predict the beginning and the end of transmembrane spans in different position so that the localization of some mutations remains uncertain. The residues G24W (hG24W) and P104L (hP98L) are localized in the first and third transmembrane spans, respectively, and R51W –R51Q (hR50Q-hR50W) in the hydrophilic matrix regions. The localization of the residues A168D (hA162D), N172K (hN166K) and S176F (hS170F) is different according different programs. In particular, TMPred and Pholyphobius placed them in the fourth transmembrane span, Phylius and Spoctopus placed the first two in the fourth transmembrane span and the third in C-terminal domain, Uniprot placed them in C-terminal domain (data not shown).

Our study provides a molecular rationale for the assignment of all the variants analyzed as pathogenic. The phenotypic analyses demonstrated the deleterious effect of the seven missense mutations studied, confirming a Sym1 role in OXPHOS metabolism and in mtDNA stability. In particular, we have demonstrated that five mutant variants, R51W (hR50W), P104L (hP98L), A168D (hA162D), N172K (hN166K) and S176F (hS170F), are completely unable to rescue the *sym1Δ* OXPHOS defect. The variants harboring the G24W (hG24W) and R51Q (hR50Q) mutations have led to a relatively milder phenotype, although the growth was strongly compromised. In agreement with our observation, the human R50Q equivalent to yeast R51Q mutation is associated with a milder phenotype [5]. In fact, in contrast to most *MPV17* gene mutations that are associated with death in infancy or early childhood, the hR50Q mutation is associated with longer survival, suggesting that this is a hypomorphic mutation [25]. Regarding to mtDNA stability, all the mutant strains showed a significant increase of *petite* colonies respect to the wild type in agreement with the defects in mtDNA maintenance observed in hepatocerebral MDDS patients.

The observed reduction of protein levels of mutated Mpv17 in patients’ cells, although detected only in two patients with the same mutation [5], has raised questions regarding stability or mistargeting of the proteins within patient cells. We then used the yeast model to try to solve this issue. Yeast can provide information about stability and localization of the mutated proteins as these can be easily tagged with the HA-tag, and, as specifically demonstrated for Sym1-HA, the binding of the tag does not interfere with the mitochondrial localization nor with mitochondrial function, allowing the use of an efficient antibody for protein detection.

All the Sym1 mutant proteins studied correctly localize into the mitochondria, indicating that none of these did compromise the mitochondrial target sequence that is still unknown. In fact, the import is not mediated by a classical N-terminus sequence subsequently removed, as previously demonstrated both for Mpv17 and Sym1 [5, 11] and the removal of N- or C-terminal segments of Sym1 did not block the import of the precursor proteins, thus excluding an essential role of these regions in the transport process and indicating that an internal targeting sequence is involved in Sym1 import [11].

To explore the other aspect, i.e. protein stability, we measured the steady state level of the seven Sym1 mutant proteins compared to the wild type protein. As depicted in Fig 4, the amount of the protein carrying the G24W (hG24W) mutation was drastically reduced, whereas the amount of the proteins carrying the R51W (hR50W), A168D (hA162D), N172K (hN166K) and S176F (hS170F) mutations are only slightly decreased compared to the wild type protein. No difference in steady state level was observed for the proteins carrying the R51Q (hR50Q) and P104L (hP98L) mutations. The results obtained and reported here favour the hypothesis that for the G24W (hG24W) protein the main molecular mechanism underlying the pathology is protein instability rather than the compromising of the high molecular-weight complex.The presence of Sym1 in a high molecular-weight complex offered another hint for investigation, the possibility that the mutations compromise the Sym1 ability to take part in a fully assembled complex, as previously demonstrated for Sym1 constructs lacking one transmembrane segment [11].

The 2D-BNGE analysis has highlighted that the protein carrying the G24W (hG24W) mutation, though quantitatively reduced, is the only capable of being part of the complex supporting our hypothesis that protein instability is the pathogenetic mechanism. All the other mutations, though at different extent, compromise the Sym1 interaction with the other complex components, as demonstrated by its inability to assemble into mature complexes and by the consistent fraction of the Sym1 mutant proteins found free or in not fully assembled complex. Despite the function of the complex is still elusive, we suggest that the pathogenicity of these mutations is related to the inability to form a fully assembled functional complex. As previously demonstrated for the homologous peroxisomal membrane protein 2 (PXMP2) that form a relatively wide channel in the mammalian peroxisomal membrane [26], Sym1 was found to form a channel with a pore size of about 1.6 nm capable of allowing the transport of large molecules, such as metabolites, across the inner mitochondrial membrane [11]. A pore-forming activity in artificial membrane was determined also for human Mpv17 protein [12]. However, we suggest that other constituents of detected complexes could be functionally relevant for the observed channel properties. The specific molecule/molecules transported by the Sym1-Mpv17 channel remains to be determined. Given the functional conservation between the different *MPV17* homologs, it has been suggested that Mpv17 forms a channel in the inner mitochondrial membrane, supplying the matrix with deoxynucleotide phosphates and/or nucleotide precursors [8].

In conclusion, based on the results here presented, we suggest that the presence of Sym1 into the high molecular weight complex is fundamental for the correct mitochondrial functionality. The specific function of the complex remains to be determined. Although it is possible that Sym1 can form oligomers, the very higher molecular weight of the complex (>600 kDa) respect that of the Sym1/MPV17 protein (20 kDa) favor the idea that Sym1 forms complexes also with other proteins. The purification of the whole complex and the identification of the Sym1/Mpv17 partner proteins could help to answer to the unsolved questions.

## Supporting information

S1 Fig Overexposure of two Western blot (WB1 and WB2) on denaturing SDS–PAGE of proteins from *sym1Δ* strains expressing the HA-tagged Sym1 recombinant variants or an empty plasmid. (TIF)

S2 Fig mRNA levels of *SYM1* wild type and mutant genes. The mRNA levels of *SYM1* wild type and mutant genes were quantified by RT-qPCR. Values are reported as mRNA level normalized respect to WT. Expression was normalized to the mRNA levels of the internal control *ACT1*. NS: not significat, *p<0.5, **p<0.01, ***p<0.001.
(TIF)

S1 Table *MPV17* pathogenic allelic variants. List of *MPV17* pathogenic mutations identified in patients, correspondent protein amino acid change in human and yeast and references. The mutations studied in this work are highlighted in yellow.
(DOC)

S2 Table Primers. List of primers used in this work.
(DOC)

S3 Table *In silico* prediction of the stability of Mpv17 mutant proteins. (DOCX)
