# Supplementary figures and images for: Pathological alleles of MPV17 modeled in the yeast Saccharomyces cerevisiae orthologous gene SYM1 reveal their inability to take part in a high molecular weight complex
Source: PLoS One. 2018 Oct 1;13(10):e0205014. doi: 10.1371/journal.pone.0205014 (PMC6166979; doi:10.1371/journal.pone.0205014)

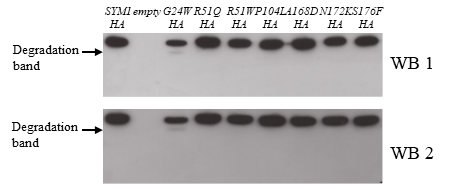

Supplement: S1 Fig — (TIF) [file pone.0205014.s001.tif]

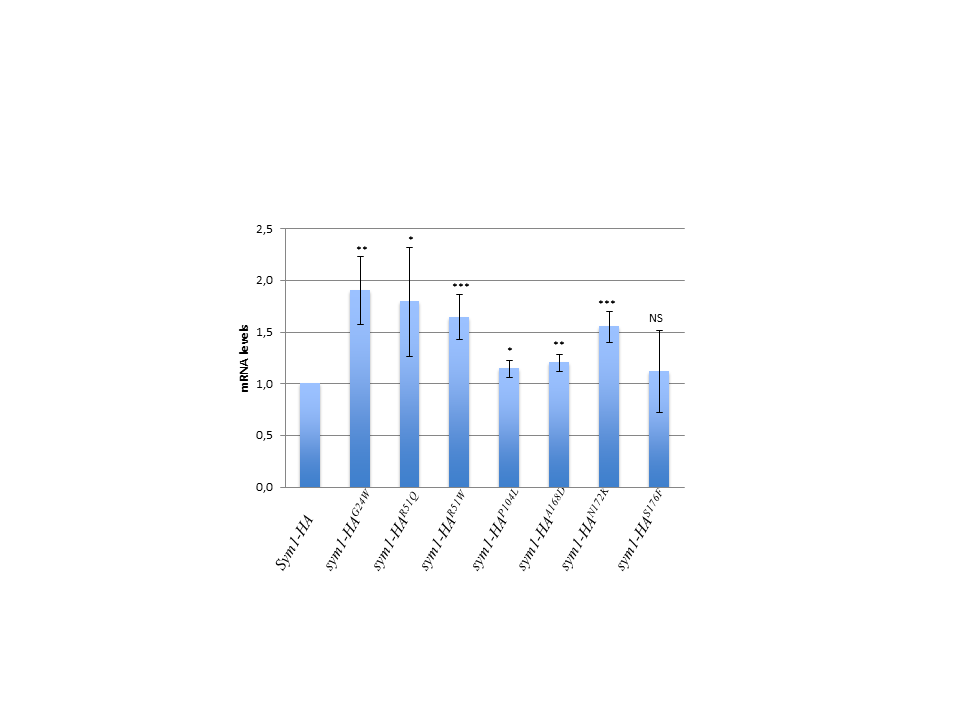

Supplement: S2 Fig — The mRNA levels of SYM1 wild type and mutant genes were quantified by RT-qPCR. Values are reported as mRNA level normalized respect to WT. Expression was normalized to the mRNA levels of the internal control ACT1. NS: not significat, *p<0.5, **p<0.01, ***p<0.001. (TIF) [file pone.0205014.s002.tif]
