# Supplementary material for: Pathological alleles of MPV17 modeled in the yeast Saccharomyces cerevisiae orthologous gene SYM1 reveal their inability to take part in a high molecular weight complex
Source: PLoS One. 2018 Oct 1;13(10):e0205014. doi: 10.1371/journal.pone.0205014 (PMC6166979; doi:10.1371/journal.pone.0205014)
Supplement: S1 Table — List of MPV17 pathogenic mutations identified in patients, correspondent protein amino acid change in human and yeast and references. The mutations studied in this work are highlighted in yellow. (DOC) [file pone.0205014.s003.doc]

| **Type of mutation** | **DNA Nucleotide Change** | **Protein Aminoacid Change** | **Protein Aminoacid Change in Yeast** | **Reference*** |
| --- | --- | --- | --- | --- |
| **Missense** | c.62T>G | p.Leu21Arg | NC | Uusimaa et al 2014 - Al Hussaini et al 2014 |
|  | c.67G>C | p.Ala23Pro | NC | Uusimaa et al 2014 |
|  | c.70G>T | p.Gly24Trp | p.Gly24Trp | Spinazzola et al 2008 |
|  | c.107A>C | p.Gln36Pro | NC | Uusimaa et al 2014 |
|  | c.122G> A | p.Arg41Gln | NC | Choi et al 2015 |
|  | c.121C>T | p.Arg41Trp | NC | Uusimaa et al 2014 - Pyal et al 2017 |
|  | c.148C>T | p.Arg50Trp | p.Arg51Trp | Spinazzola et al 2006 - Wong et al 2007 - Vilarinho et al 2014 |
|  | c.149G>A | p.Arg50Gln | p.Arg51Gln | Spinazzola et al 2006 - Karadimas et al 2006 - El-Hattab et al 2010 |
|  | c.191C>G | p.Pro64Arg | NC | Uusimaa et al 2014 - Piekutowska-Abramczuk 2014 |
|  | c.197T>A | p.Val66Glu | NC | Kim et al 2016 |
|  | c.262A>G | p.Lys88Glu | NC | El-Hattab et al 2010 |
|  | c.263A>T | p.Lys88Met | NC | Garone et al 2012 |
|  | c.265A>T | p.Met89Leu | NC | Garone et al 2012 |
|  | c.278A>C | p.Gln93Pro | p.Gln99Pro | Uusimaa et al 2014 - Sarkhy et al 2014 - Al Hussaini et al 2014 |
|  | c.280G>T c.280G>C | p.Gly94Arg | NC | El-Hattab et al 2010 - Kim et al 2016 |
|  | c.293C>T | p.Pro98Leu | p.Pro104Leu | El-Hattab et al 2010 - Blakely 2012 et al - Uusima et al 2014 - Bijarnia-Mahay et al 2014 - Kim et al 2016 |
|  | c.485C>A | p.Ala162Asp | p.Ala168Asp | El-Hattab et al 2010 |
|  | c.498C>A | p.Asn166Lys | p.Asn172Lys | Spinazzola et al 2006 - Sarzi et al 2007 |
|  | c.509C>T | p.Ser170Phe | P.Ser176Phe | Kaji et al 2009 |
| **Nonsense** | c.130C>T | p.Gln44Ter |  | Uusimaa et al 2014 |
|  | c.206G>A | p.Trp69Ter |  | Wong et al 2007 |
|  | c.359G>A | p.Trp120Ter |  | Spinazzola et al 2008 |
|  | c.408T>G | p.Tyr136Ter |  | Bijarnia-Mahay et al 2014 |
|  | c.428T>G | p.Leu143Ter |  | Garone et al 2012 |
| **In-frame deletion** | c.234_242del9 | p.Gly79_Thr81del |  | Wong et al 2007 |
|  | c.263_265del3 | p.Lys88del |  | Wong et al 2007 - El-Hattab et al 2010 |
|  | c.271_273del3 | p.Leu91del |  | El-Hattab et al 2010 |
| **Frame shift deletion** | c.135delA | p.Glu45AspfsTer8 |  | Uusimaa et al 2014 |
|  | c.116-141del26 | p.Arg41ProfsTer24 |  | Spinazzola et al 2006 |
| **Insertion** | c.22_23insC | p.Gln8ProfsTer24 |  | El-Hattab et al 2010 |
|  | c.451_452insC | p.Leu151ProfsTer39 |  | Kaji et al 2009 - Kim et al 2016 |
| **Splicing site** | c.IVS3+1G>T |  |  | Alsaman et al 2012 |
|  | c.70+5G>A |  |  | Navarro-Sastre et al 2008 - Navarro-Sastre et al 2010 - Navarro-Sastre et al 2012 |
|  | c.186+2T>C |  |  | El-Hattab et al 2010 - Nogueira et al., 2012 |
|  | c.279+1G>T |  |  | Uusimaa et al 2014 - Al Hussaini et al 2014 |
|  | c.461+1G>C |  |  | Uusimaa et al 2014 |
| **Large deletion** | 1.57 Kb deletion spanning exon 8 |  |  | Spinazzola et al 2008 - El-Hattab et al 2010 |
|  | exons 3-8 deletion |  |  | Uusimaa et al 2014 |

*** Refererences:**

Al-Hussaini A, Faqeih E, El-Hattab AW, Alfadhel M, Asery A, Alsaleem B, et al. [Clinical and molecular characteristics of mitochondrial DNA depletion syndrome associated with neonatal cholestasis and liver failure.](https://www.ncbi.nlm.nih.gov/pubmed/24321534) *J. Pediatr.* 2014;164: 553-559.

[AlSaman A](https://www.ncbi.nlm.nih.gov/pubmed/?term=AlSaman A%5BAuthor%5D&cauthor=true&cauthor_uid=22824774), [Tomoum H](https://www.ncbi.nlm.nih.gov/pubmed/?term=Tomoum H%5BAuthor%5D&cauthor=true&cauthor_uid=22824774), [Invernizzi F](https://www.ncbi.nlm.nih.gov/pubmed/?term=Invernizzi F%5BAuthor%5D&cauthor=true&cauthor_uid=22824774), [Zeviani M](https://www.ncbi.nlm.nih.gov/pubmed/?term=Zeviani M%5BAuthor%5D&cauthor=true&cauthor_uid=22824774). Hepatocerebral form of mitochondrial DNA depletion syndrome due to mutation in MPV17 gene. [*Saudi J. Gastroenterol.*](https://www.ncbi.nlm.nih.gov/pubmed/?term=alsaman+and+mpv17) 2012;18: 285-289.

[Bijarnia-Mahay S](https://www.ncbi.nlm.nih.gov/pubmed/?term=Bijarnia-Mahay S%5BAuthor%5D&cauthor=true&cauthor_uid=25129007), [Mohan N](https://www.ncbi.nlm.nih.gov/pubmed/?term=Mohan N%5BAuthor%5D&cauthor=true&cauthor_uid=25129007), [Goyal D](https://www.ncbi.nlm.nih.gov/pubmed/?term=Goyal D%5BAuthor%5D&cauthor=true&cauthor_uid=25129007), [Verma IC](https://www.ncbi.nlm.nih.gov/pubmed/?term=Verma IC%5BAuthor%5D&cauthor=true&cauthor_uid=25129007). Mitochondrial DNA depletion syndrome causing liver failure. [*Indian Pediatr.*](https://www.ncbi.nlm.nih.gov/pubmed/?term=sunita+bijarnia+and+mpv17) 2014;51: 666-668.

Bitting CP, Hanson JA. [Navajo Neurohepatopathy : A Case Report and Literature Review Emphasizing Clinicopathologic Diagnosis.](https://www.ncbi.nlm.nih.gov/pubmed/28209105) *Acta Gastroenterol. Belg*. 2016;79: 463-469.

[Blakely EL](https://www.ncbi.nlm.nih.gov/pubmed/?term=Blakely EL%5BAuthor%5D&cauthor=true&cauthor_uid=22508010), [Butterworth A](https://www.ncbi.nlm.nih.gov/pubmed/?term=Butterworth A%5BAuthor%5D&cauthor=true&cauthor_uid=22508010), [Hadden RD](https://www.ncbi.nlm.nih.gov/pubmed/?term=Hadden RD%5BAuthor%5D&cauthor=true&cauthor_uid=22508010), [Bodi I](https://www.ncbi.nlm.nih.gov/pubmed/?term=Bodi I%5BAuthor%5D&cauthor=true&cauthor_uid=22508010), [He L](https://www.ncbi.nlm.nih.gov/pubmed/?term=He L%5BAuthor%5D&cauthor=true&cauthor_uid=22508010), [McFarland R](https://www.ncbi.nlm.nih.gov/pubmed/?term=McFarland R%5BAuthor%5D&cauthor=true&cauthor_uid=22508010), et al. MPV17 mutation causes neuropathy and leukoencephalopathy with multiple mtDNA deletions in muscle. [*Neuromuscul. Disord.*](https://www.ncbi.nlm.nih.gov/pubmed/?term=blakely+and+mpv17) 2012;22: 587-591.

Choi YR, Hong YB, Jung SC, Lee JH, Kim YJ, Park HJ, et al. [A novel homozygous MPV17 mutation in two families with axonal sensorimotor polyneuropathy.](https://www.ncbi.nlm.nih.gov/pubmed/26437932) *BMC Neurol.* 2015;5: 15-179.

[El-Hattab AW](https://www.ncbi.nlm.nih.gov/pubmed/?term=El-Hattab AW%5BAuthor%5D&cauthor=true&cauthor_uid=20074988), [Li FY](https://www.ncbi.nlm.nih.gov/pubmed/?term=Li FY%5BAuthor%5D&cauthor=true&cauthor_uid=20074988), [Schmitt E](https://www.ncbi.nlm.nih.gov/pubmed/?term=Schmitt E%5BAuthor%5D&cauthor=true&cauthor_uid=20074988), [Zhang S](https://www.ncbi.nlm.nih.gov/pubmed/?term=Zhang S%5BAuthor%5D&cauthor=true&cauthor_uid=20074988), [Craigen WJ](https://www.ncbi.nlm.nih.gov/pubmed/?term=Craigen WJ%5BAuthor%5D&cauthor=true&cauthor_uid=20074988), [Wong LJ](https://www.ncbi.nlm.nih.gov/pubmed/?term=Wong LJ%5BAuthor%5D&cauthor=true&cauthor_uid=20074988). MPV17-associated hepatocerebral mitochondrial DNA depletion syndrome: new patients and novel mutations. [*Mol. Genet. Metab.*](https://www.ncbi.nlm.nih.gov/pubmed/20074988) 2010;99: 300-308.

[Garone C](https://www.ncbi.nlm.nih.gov/pubmed/?term=Garone C%5BAuthor%5D&cauthor=true&cauthor_uid=22964873), [Rubio JC](https://www.ncbi.nlm.nih.gov/pubmed/?term=Rubio JC%5BAuthor%5D&cauthor=true&cauthor_uid=22964873), [Calvo SE](https://www.ncbi.nlm.nih.gov/pubmed/?term=Calvo SE%5BAuthor%5D&cauthor=true&cauthor_uid=22964873), [Naini A](https://www.ncbi.nlm.nih.gov/pubmed/?term=Naini A%5BAuthor%5D&cauthor=true&cauthor_uid=22964873), [Tanji K](https://www.ncbi.nlm.nih.gov/pubmed/?term=Tanji K%5BAuthor%5D&cauthor=true&cauthor_uid=22964873), [Dimauro S](https://www.ncbi.nlm.nih.gov/pubmed/?term=Dimauro S%5BAuthor%5D&cauthor=true&cauthor_uid=22964873), et al. 2012 MPV17 Mutations Causing Adult-Onset Multisystemic Disorder With Multiple Mitochondrial DNA Deletions. [*Arch. Neurol.*](https://www.ncbi.nlm.nih.gov/pubmed/?term=garone+and+mpv17) , **69** , 1648-1651.

[Kaji S](https://www.ncbi.nlm.nih.gov/pubmed/?term=Kaji S%5BAuthor%5D&cauthor=true&cauthor_uid=19520594), [Murayama K](https://www.ncbi.nlm.nih.gov/pubmed/?term=Murayama K%5BAuthor%5D&cauthor=true&cauthor_uid=19520594), [Nagata I](https://www.ncbi.nlm.nih.gov/pubmed/?term=Nagata I%5BAuthor%5D&cauthor=true&cauthor_uid=19520594), [Nagasaka H](https://www.ncbi.nlm.nih.gov/pubmed/?term=Nagasaka H%5BAuthor%5D&cauthor=true&cauthor_uid=19520594), [Takayanagi M](https://www.ncbi.nlm.nih.gov/pubmed/?term=Takayanagi M%5BAuthor%5D&cauthor=true&cauthor_uid=19520594), [Ohtake A](https://www.ncbi.nlm.nih.gov/pubmed/?term=Ohtake A%5BAuthor%5D&cauthor=true&cauthor_uid=19520594), et al. Fluctuating liver functions in siblings with MPV17 mutations and possible improvement associated with dietary and pharmaceutical treatments targeting respiratory chain complex II. [*Mol. Genet. Metab.*](https://www.ncbi.nlm.nih.gov/pubmed/?term=Kaji+and+mpv17) 2009;97: 292-296.

[Karadimas CL](https://www.ncbi.nlm.nih.gov/pubmed/?term=Karadimas CL%5BAuthor%5D&cauthor=true&cauthor_uid=16909392), [Vu TH](https://www.ncbi.nlm.nih.gov/pubmed/?term=Vu TH%5BAuthor%5D&cauthor=true&cauthor_uid=16909392), [Holve SA](https://www.ncbi.nlm.nih.gov/pubmed/?term=Holve SA%5BAuthor%5D&cauthor=true&cauthor_uid=16909392), [Chronopoulou P](https://www.ncbi.nlm.nih.gov/pubmed/?term=Chronopoulou P%5BAuthor%5D&cauthor=true&cauthor_uid=16909392), [Quinzii C](https://www.ncbi.nlm.nih.gov/pubmed/?term=Quinzii C%5BAuthor%5D&cauthor=true&cauthor_uid=16909392), [Johnsen SD](https://www.ncbi.nlm.nih.gov/pubmed/?term=Johnsen SD%5BAuthor%5D&cauthor=true&cauthor_uid=16909392), et al. Navajo neurohepatopathy is caused by a mutation in the MPV17 gene. [*Am. J. Hum. Genet.*](https://www.ncbi.nlm.nih.gov/pubmed/16909392) 2006;79: 544-548.

Kim J, Kang E, Kim Y, Kim JM, Lee BH, Murayama K, et al. [MPV17 mutations in patients with hepatocerebral mitochondrial DNA depletion syndrome.](https://www.ncbi.nlm.nih.gov/pubmed/27536553) *Mol. Genet. Metab. Rep.* 2016;8: 74-76.

Navarro-Sastre A, García-Silva MT, Martín-Hernández E, Lluch M, Briones P, Ribes A. [Functional splicing assay supporting that c.70 + 5G &gt; A mutation in the MPV17 gene is disease causing.](https://www.ncbi.nlm.nih.gov/pubmed/20614188) *J. Inherit. Metab. Dis.* 2010;33.

Navarro-Sastre A, Martín-Hernández E, Campos Y, Quintana E, Medina E, de Las Heras RS, et al. Lethal hepatopathy and leukodystrophy caused by a novel mutation in MPV17 gene: description of an alternative MPV17 spliced form. *Mol. Genet. Metab.* 2008;94: 234-239.

Navarro-Sastre A, Tort F, Garcia-Villoria J, Pons MR, Nascimento A, Colomer J, et al. [Mitochondrial DNA depletion syndrome: new descriptions and the use of citrate synthase as a helpful tool to better characterise the patients.](https://www.ncbi.nlm.nih.gov/pubmed/22980518) *Mol. Genet. Metab.* 2012;107:409-415.

[Nogueira C](https://www.ncbi.nlm.nih.gov/pubmed/?term=Nogueira C%5BAuthor%5D&cauthor=true&cauthor_uid=23137571), [de Souza CF](https://www.ncbi.nlm.nih.gov/pubmed/?term=de Souza CF%5BAuthor%5D&cauthor=true&cauthor_uid=23137571), [Husny A](https://www.ncbi.nlm.nih.gov/pubmed/?term=Husny A%5BAuthor%5D&cauthor=true&cauthor_uid=23137571), [Derks TG](https://www.ncbi.nlm.nih.gov/pubmed/?term=Derks TG%5BAuthor%5D&cauthor=true&cauthor_uid=23137571), [Santorelli FM](https://www.ncbi.nlm.nih.gov/pubmed/?term=Santorelli FM%5BAuthor%5D&cauthor=true&cauthor_uid=23137571), [Vilarinho L](https://www.ncbi.nlm.nih.gov/pubmed/?term=Vilarinho L%5BAuthor%5D&cauthor=true&cauthor_uid=23137571). MPV17: fatal hepatocerebral presentation in a Brazilian infant. [*Mol. Genet. Metab.*](https://www.ncbi.nlm.nih.gov/pubmed/23137571) 2012;107: 764.

Piekutowska-Abramczuk D, Pronicki M, Strawa K, Karkucińska-Więckowska A, Szymańska-Dębińska T, Fidziańska A, et al. [Novel c.191C>G (p.Pro64Arg) MPV17 mutation identified in two pairs of unrelated Polish siblings with mitochondrial hepatoencephalopathy.](https://www.ncbi.nlm.nih.gov/pubmed/23829229) *Clin. Genet.* 2014;85: 573-577.

[Pyal A](https://www.ncbi.nlm.nih.gov/pubmed/?term=Pyal A%5BAuthor%5D&cauthor=true&cauthor_uid=28673863), [Paramasivam A](https://www.ncbi.nlm.nih.gov/pubmed/?term=Paramasivam A%5BAuthor%5D&cauthor=true&cauthor_uid=28673863), [Meena AK](https://www.ncbi.nlm.nih.gov/pubmed/?term=Meena AK%5BAuthor%5D&cauthor=true&cauthor_uid=28673863), [Bhavana VB](https://www.ncbi.nlm.nih.gov/pubmed/?term=Bhavana VB%5BAuthor%5D&cauthor=true&cauthor_uid=28673863), [Thangaraj K](https://www.ncbi.nlm.nih.gov/pubmed/?term=Thangaraj K%5BAuthor%5D&cauthor=true&cauthor_uid=28673863). MPV17 hepatocerebral mitochondrial DNA depletion syndrome presenting as acute flaccid paralysis - A case report. [*Mitochondrion.*](https://www.ncbi.nlm.nih.gov/pubmed/?term=MPV17+hepatocerebral+mitochondrial+DNA+depletion+syndrome+presenting+as+acute+flaccid+paralysis+-+A+case+report.+Mitochondrion.)2017;37: 41-45.

[Sarkhy AA](https://www.ncbi.nlm.nih.gov/pubmed/?term=Sarkhy AA%5BAuthor%5D&cauthor=true&cauthor_uid=24894789), [Al-Sunaid A](https://www.ncbi.nlm.nih.gov/pubmed/?term=Al-Sunaid A%5BAuthor%5D&cauthor=true&cauthor_uid=24894789), [Abdullah A](https://www.ncbi.nlm.nih.gov/pubmed/?term=Abdullah A%5BAuthor%5D&cauthor=true&cauthor_uid=24894789), [AlFadhel M](https://www.ncbi.nlm.nih.gov/pubmed/?term=AlFadhel M%5BAuthor%5D&cauthor=true&cauthor_uid=24894789), [Eiyad W](https://www.ncbi.nlm.nih.gov/pubmed/?term=Eiyad W%5BAuthor%5D&cauthor=true&cauthor_uid=24894789). A novel MPV17 gene mutation in a Saudi infant causing fatal progressive liver failure. [*Ann. Saudi Med.*](https://www.ncbi.nlm.nih.gov/pubmed/?term=sarkhy+and+mpv17) 2014;34: 175-178.

[Sarzi E](https://www.ncbi.nlm.nih.gov/pubmed/?term=Sarzi E%5BAuthor%5D&cauthor=true&cauthor_uid=17452231), [Bourdon A](https://www.ncbi.nlm.nih.gov/pubmed/?term=Bourdon A%5BAuthor%5D&cauthor=true&cauthor_uid=17452231), [Chrétien D](https://www.ncbi.nlm.nih.gov/pubmed/?term=Chrétien D%5BAuthor%5D&cauthor=true&cauthor_uid=17452231), [Zarhrate M](https://www.ncbi.nlm.nih.gov/pubmed/?term=Zarhrate M%5BAuthor%5D&cauthor=true&cauthor_uid=17452231), [Corcos J](https://www.ncbi.nlm.nih.gov/pubmed/?term=Corcos J%5BAuthor%5D&cauthor=true&cauthor_uid=17452231), [Slama A](https://www.ncbi.nlm.nih.gov/pubmed/?term=Slama A%5BAuthor%5D&cauthor=true&cauthor_uid=17452231), et al. Mitochondrial DNA depletion is a prevalent cause of multiple respiratory chain deficiency in childhood. [*J. Pediatr.*](https://www.ncbi.nlm.nih.gov/pubmed/17452231) 2007;150: 531-534.

Spinazzola A, Santer R, Akman OH, Tsiakas K, Schaefer H, Ding X, et al. Hepatocerebral form of mitochondrial DNA depletion syndrome: novel MPV17 mutations. *Arch. Neurol.* 2008;65: 1108-1113.

Uusimaa J, Evans J, Smith C, Butterworth A, Craig K, Ashley N, et al. [Clinical, biochemical, cellular and molecular characterization of mitochondrial DNA depletion syndrome due to novel mutations in the MPV17 gene.](https://www.ncbi.nlm.nih.gov/pubmed/23714749) *Eur. J. Hum. Genet.* 2014;22: 184-191.

Vilarinho S, Choi M, Jain D, Malhotra A, Kulkarni S, Pashankar D, et al. [Individual exome analysis in diagnosis and management of paediatric liver failure of indeterminate aetiology.](https://www.ncbi.nlm.nih.gov/pubmed/25016221) *J. Hepatol.* 2014;61: 1056-1063.

Wong LJ, Brunetti-Pierri N, Zhang Q, Yazigi N, Bove KE, Dahms BB, et al. [Mutations in the MPV17 gene are responsible for rapidly progressive liver failure in infancy.](https://www.ncbi.nlm.nih.gov/pubmed/17694548) *Hepatology.* 2007;46: 1218-1227.
