# Supplementary material for: Pathological alleles of MPV17 modeled in the yeast Saccharomyces cerevisiae orthologous gene SYM1 reveal their inability to take part in a high molecular weight complex
Source: PLoS One. 2018 Oct 1;13(10):e0205014. doi: 10.1371/journal.pone.0205014 (PMC6166979; doi:10.1371/journal.pone.0205014)
Supplement: S2 Table — List of primers used in this work. (DOC) [file pone.0205014.s004.doc]

| **Amino acid change** | **Mutagenic primers^a^** |
| --- | --- |
| G24W | Fw: CTACGAATGCGATAATGACAtGgGCGCTATTTGGAATTGGTGATG Rv: CATCACCAATTCCAAATAGCGCcCaTGTCATTATCGCATTCGTAG |
| R51Q | Fw: AAACAAGGGTTATGATTATAAAcaaACAGCTAGGGCTGTCATCTATG  Rv: CATAGATGACAGCCCTAGCTGTttgTTTATAATCATAACCCTTGTTT |
| R51W | Fw: AAACAAGGGTTATGATTATAAAtGGACAGCTAGGGCTGTCATCTATG  Rv: CATAGATGACAGCCCTAGCTGTCCaTTTATAATCATAACCCTTGTTT |
| P104L | Fw: CGATCAATTGGCGTTTGCGttGCTAGGTTTGCCATTTTATTTCACC  Rv: GGTGAAATAAAATGGCAAACCTAGCaaCGCAAACGCCAATTGATCG |
| A168D | Fw: GGTTACTAGCTGTTAATGTCGTTGatATATTTTGGAACACTTACTTATC  Rv: GATAAGTAAGTGTTCCAAAATATatCAACGACATTAACAGCTAGTAACC |
| N172K | Fw: TGTCGTTGCAATATTTTGGAAgACTTACTTATCTTATAAAAACTC  Rv: GAGTTTTTATAAGATAAGTAAGTcTTCCAAAATATTGCAACGACA |
| S176F | Fw: GGAACACTTACTTATtTTATAAAAACTCAAAGGTTATGGAGAAAG  Rv: CTTTCTCCATAACCTTTGAGTTTTTATAAaATAAGTAAGTGTTCC |
| qSYM1 | Fw: CGCCGCTAGGTTTGCCATTT  Rv: AGTGTAGGCCACCATTGCTCT |
| ACT1q | Fw: GTATGTGTAAAGCCGGTTTTG  Rv: CATGATACCTTGGTGTCTTGG |

^a^  Nucleotide changes are in lowercase
