# Supplementary material for: Pathological alleles of MPV17 modeled in the yeast Saccharomyces cerevisiae orthologous gene SYM1 reveal their inability to take part in a high molecular weight complex
Source: PLoS One. 2018 Oct 1;13(10):e0205014. doi: 10.1371/journal.pone.0205014 (PMC6166979; doi:10.1371/journal.pone.0205014)
Supplement: S3 Table — (DOCX) [file pone.0205014.s005.docx]

| **Mpv17 substitution** | **I-mutant 2.0** | **Mupro** | **Istable** | **INPS-MD** |
| --- | --- | --- | --- | --- |
| G24W | Decreased | Decreased | Increased | Decreased |
| R50Q | Neutral | Decreased | Increased | Decreased |
| R50W | Neutral | Decreased | Increased | Decreased |
